# Underrepresentation of Patients From Racial and Ethnic Minorities in Immunotherapy Clinical Trials for Non-Small Cell Lung Cancer

**DOI:** 10.1016/j.atssr.2026.03.019

**Published:** 2026-05-20

**Authors:** Deepti Srinivasan, Camille Mathey-Andrews, Shivaek Venkateswaran, Ashley Sanchez, Alexandra L. Potter, Arian Mansur, Lana Schumacher, Chi-Fu Jeffrey Yang

**Affiliations:** 1 Division of Thoracic Surgery, Department of Surgery, Massachusetts General Hospital, Boston, Massachusetts; 2 Division of Thoracic Surgery, Department of Surgery, Tufts Medical Center, Boston, Massachusetts

## Abstract

**Background:**

Landmark immunotherapy trials have transformed treatment of non-small cell lung cancer (NSCLC), yet underrepresentation of racial and ethnic minorities threatens the generalizability of trial findings. This study evaluated disparities in receipt of immunotherapy during clinical trial periods preceding regulatory approval.

**Methods:**

Using the National Cancer Database, we assessed receipt of immunotherapy by patients diagnosed with metastatic NSCLC between 2004 and 2015 and resectable stage II-IIIB NSCLC between 2004 and 2021, corresponding to immunotherapy clinical trial periods for metastatic and resectable disease, respectively. Multivariable adjusted logistic regression was used to evaluate associations between race and ethnicity and receipt of immunotherapy. Additional sensitivity analyses were performed among subgroups of patients with insurance, who reside in high-income areas, received treatment at academic facilities, have no comorbidities, and received chemotherapy.

**Results:**

In multivariable analyses of patients with metastatic disease, Black (odds ratio [OR], 0.81; 95% CI, 0.76-0.88), Asian (OR, 0.84; 95% CI, 0.74-0.95), and Hispanic (OR, 0.79; 95% CI, 0.71-0.89) patients were significantly less likely than non-Hispanic White patients to receive immunotherapy. Disparities persisted in sensitivity analyses, including of insured patients treated at academic centers in high-income areas. Notably, Black patients residing in the highest income areas remained less likely to receive immunotherapy than White patients residing in the lowest income areas (OR, 0.63; 95% CI, 0.42-0.96). Similar disparities were observed in the neoadjuvant setting.

**Conclusions:**

Patients from racial and ethnic minorities were significantly less likely to receive immunotherapy during clinical trial periods for NSCLC, even in populations with comparable access to care. These findings emphasize persistent inequities in early access to novel therapies and the need for targeted strategies to improve trial representation.

**In Short**

▪ Racial and ethnic minority patients were significantly less likely to receive immunotherapy during clinical trial periods for non-small cell lung cancer.
▪ Disparities persisted even for insured patients treated at academic centers and residing in high-income areas.
▪ These findings highlight structural inequities in early access to novel cancer therapies and underscore the need for improved representation in future clinical trials.

During the past decade, immunotherapy has transformed the treatment of non-small cell lung cancer (NSCLC). Randomized trials demonstrated survival benefits in metastatic disease and, more recently, in resectable disease, leading to respective Food and Drug Administration approvals in 2015 and 2021-2022.^1^, ^2^, ^3^, ^4^ Despite these advances, long-standing concerns remain about the underrepresentation of racial and ethnic minority populations in lung cancer clinical trials, raising questions about the generalizability of trial-derived evidence.

Underreporting and underrepresentation of racial and ethnic minorities in clinical trials are pervasive across oncology. In landmark trials conducted between 2010 and 2018, race was comprehensively reported in <10%, and when race was reported, Black and Hispanic patients were substantially underrepresented relative to disease incidence.^5^ Whether similar disparities characterized the clinical trial eras that led to immunotherapy approvals in NSCLC remains incompletely understood.

The objective of this study was to characterize racial and ethnic disparities in receipt of immunotherapy during clinical trial periods for NSCLC using a large national database, recognizing receipt of immunotherapy during these periods as a proxy for trial participation.

## Patients and Methods

### Data Source and Study Design

This retrospective study was approved by the Massachusetts General Hospital Institutional Review Board (IRB #2020P004110). Data were obtained from the National Cancer Database, which captures approximately 65% of newly diagnosed lung cancers in the United States across Commission on Cancer–accredited institutions.^6^

Two cohorts were identified: patients diagnosed with metastatic NSCLC between 2004 and 2015 and patients diagnosed with resectable stage II-IIIB NSCLC between 2004 and 2021, corresponding to periods preceding Food and Drug Administration approvals of immunotherapy in each setting. Staging was harmonized to American Joint Committee on Cancer staging manual eighth edition criteria.^7^

Receipt of immunotherapy was assessed within each cohort. Patients who died before treatment or refused immunotherapy were excluded. In the resectable cohort, immunotherapy administered within 6 months before surgery was classified as neoadjuvant. To align with original trial eligibility, patients receiving neoadjuvant radiation were excluded.

Race and ethnicity were categorized as non-Hispanic White, Black, Hispanic, Asian, or Native American. Patients with unknown race or ethnicity were excluded.

### Statistical Analyses

Baseline characteristics were compared by Wilcoxon rank sum and *χ*^2^ tests. Multivariable adjusted logistic regression evaluated associations between race and ethnicity and receipt of immunotherapy, adjusting for age, sex, histologic type, comorbidity burden, insurance status, facility type, income, education, and year of diagnosis. A case complete analysis was used to address missing data. Missing values are reported in Supplemental Tables 2 and 3.

Prespecified sensitivity analyses examined insured patients treated at academic centers, patients residing in high-income areas, patients without comorbidities, and patients receiving chemotherapy. Analyses were repeated for the resectable cohort. Statistical analyses were performed with Stata 18.0 software (StataCorp).

## Results

Of 8663 patients with metastatic NSCLC who received immunotherapy during the clinical trial period, 82.3% were White, 10.1% Black, 4.0% Hispanic, and 3.3% Asian. Patients who received immunotherapy were more frequently treated at academic facilities and were more likely to reside in higher income areas compared with those who did not receive immunotherapy (Table). Receipt of immunotherapy varied by race and ethnicity across all study years.

**Table:** Baseline Characteristics of Patients With Metastatic NSCLC Diagnosed in the Clinical Trial Period Between 2004 and 2015

Variable	No Immunotherapy (N = 339,212)	Received Immunotherapy (N = 8663)	*P*
Age, y	68 (60-76)	65 (58-72)	<.001
Sex			<.001
Male	185,389 (54.7)	4539 (52.4)	
Female	153,823 (45.3)	4124 (47.6)	
Race/ethnicity			<.001
Non-Hispanic White	264,448 (78.0)	7127 (82.3)	
Hispanic	26,054 (7.7)	344 (4.0)	
Black	38,717 (11.4)	877 (10.1)	
Native American	799 (0.2)	28 (0.3)	
Asian	9194 (2.7)	287 (3.3)	
Charlson/Deyo comorbidity score			<.001
0	207,701 (61.2)	5828 (67.3)	
1	87,800 (25.9)	2102 (24.3)	
2	30,527 (9.0)	541 (6.2)	
3+	13,184 (3.9)	192 (2.2)	
Histology			<.001
Adenocarcinoma	220,418 (65.0)	7474 (86.3)	
Squamous cell carcinoma	83,146 (24.5)	730 (8.4)	
Large cell carcinoma	14,298 (4.2)	132 (1.5)	
Adenosquamous carcinoma	4911 (1.4)	79 (0.9)	
Carcinoid	10,413 (3.1)	101 (1.2)	
Bronchioalveolar carcinoma	6026 (1.8)	147 (1.7)	
Insurance type			<.001
No insurance	14,551 (4.3)	245 (2.8)	
Private	96,690 (28.5)	3291 (38.0)	
Medicaid	24,501 (7.2)	624 (7.2)	
Medicare	198,868 (58.6)	4390 (50.7)	
Other government	4602 (1.4)	113 (1.3)	
Facility type			<.001
Community cancer program	30,118 (8.9)	592 (6.8)	
Comprehensive community program	145,351 (42.8)	3663 (42.3)	
Academic/research program	95,194 (28.1)	2772 (32.0)	
Integrated network cancer program	68,549 (20.2)	1636 (18.9)	
ZIP Code income quartile			<.001
1	69,981 (20.6)	1601 (18.5)	
2	83,351 (24.6)	1985 (22.9)	
3	86,341 (25.5)	2160 (24.9)	
4	99,539 (29.3)	2917 (33.7)	
ZIP Code education quartile			<.001
1	67,808 (20.0)	1595 (18.4)	
2	95,689 (28.2)	2316 (26.7)	
3	105,150 (31.0)	2568 (29.6)	
4	70,565 (20.8)	2184 (25.2)	
Year of diagnosis	2011 (2008-2013)	2014 (2013-2015)	<.001
Received chemotherapy	179,397 (62.8)	7946 (92.5)	<.001

Categorical variables are presented as number (percentage). Continuous variables are presented as median (interquartile range).

NSCLC, non-small cell lung cancer.

**Figure 1:**
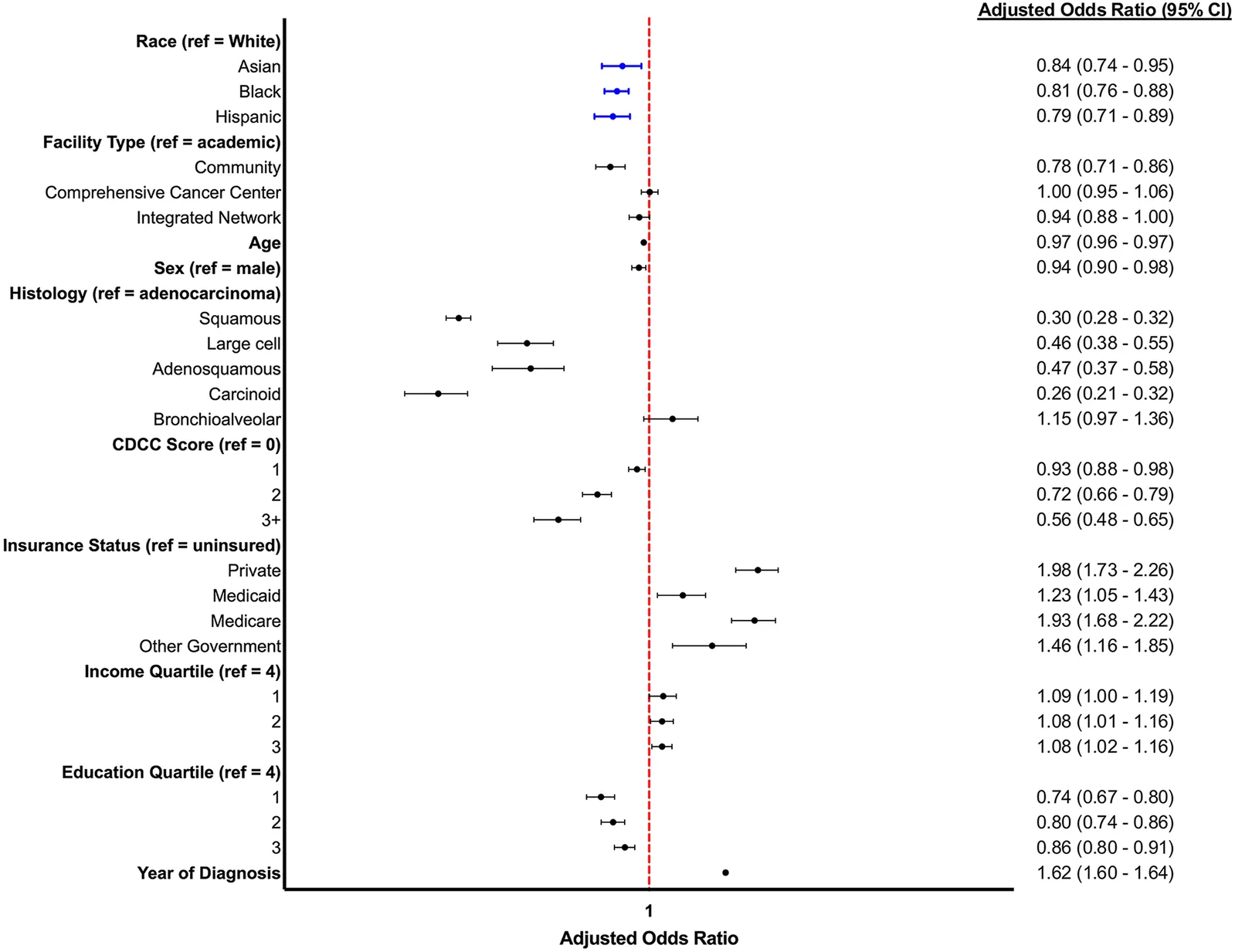
Multivariable adjusted logistic regression evaluating independent predictors of the receipt of immunotherapy for patients diagnosed with metastatic non-small cell lung cancer between 2004 and 2015. (CDCC, Charlson/Deyo comorbidity class; ref, reference.)

In multivariable adjusted analysis, Black (odds ratio [OR], 0.81; 95% CI ,0.76-0.88), Asian (OR, 0.84; 95% CI, 0.74-0.95), and Hispanic (OR, 0.79; 95% CI, 0.71-0.89) patients were less likely than non-Hispanic White patients to receive immunotherapy after adjustment for demographic, clinical, and socioeconomic factors (Figure 1).

**Figure 2:**
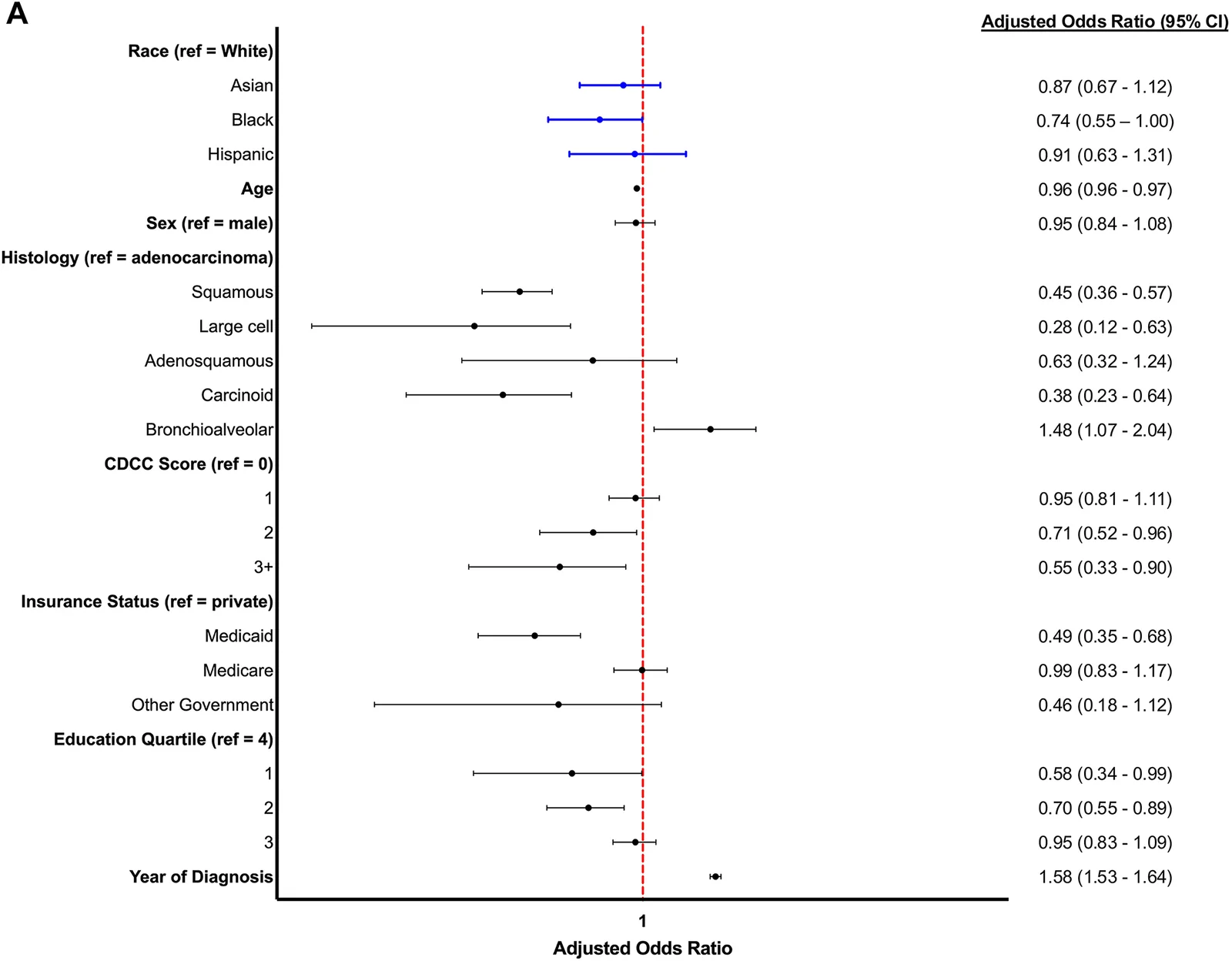

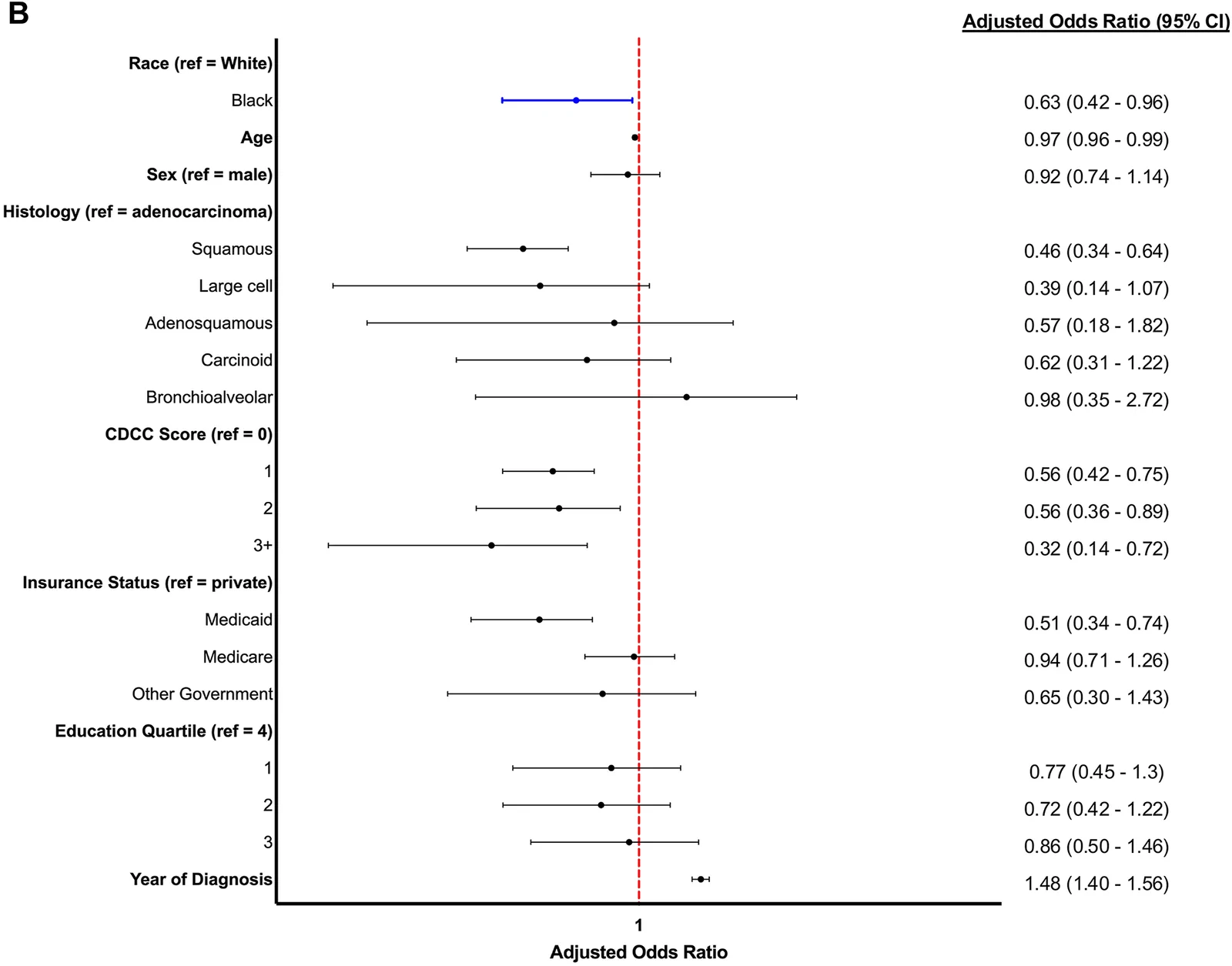

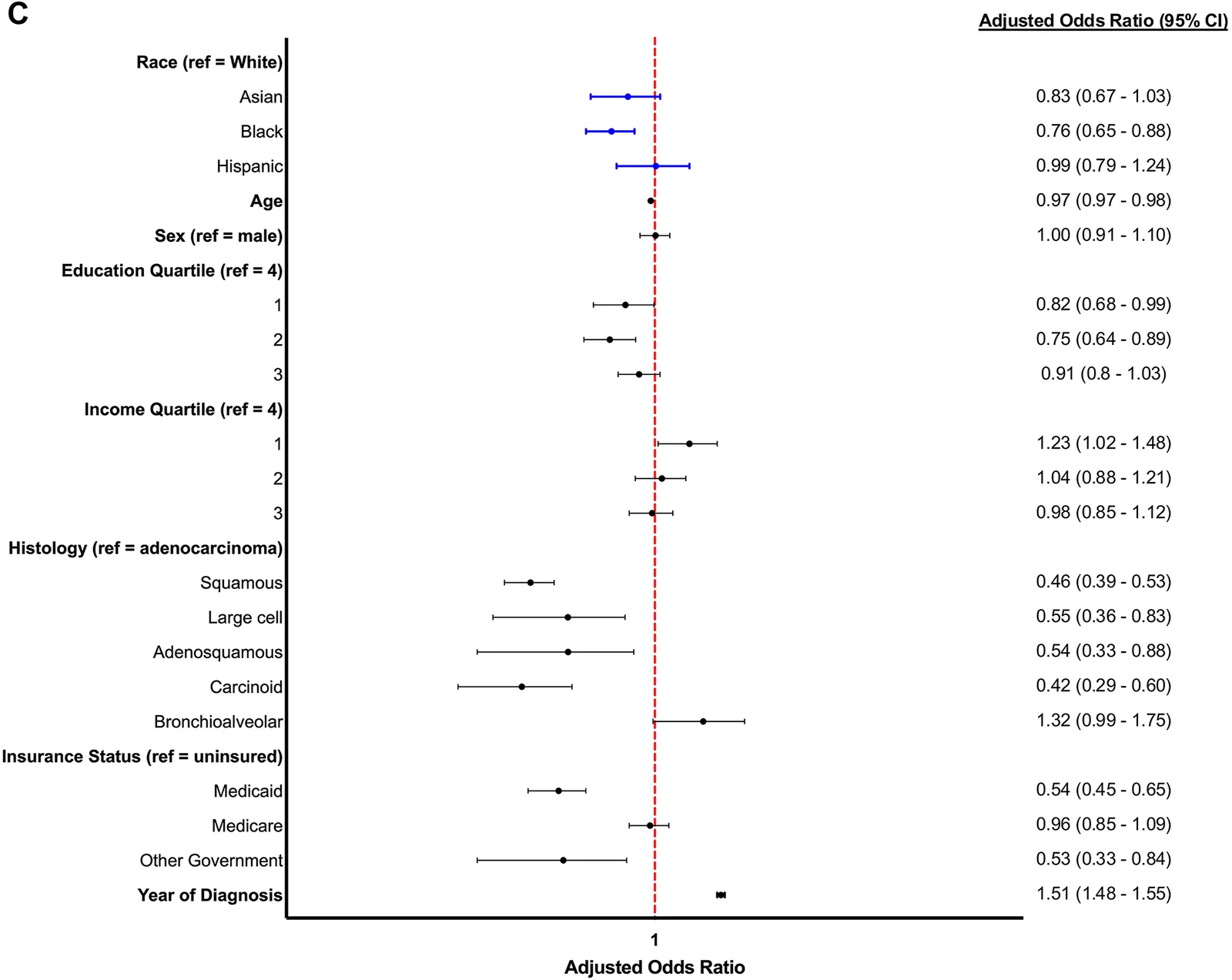

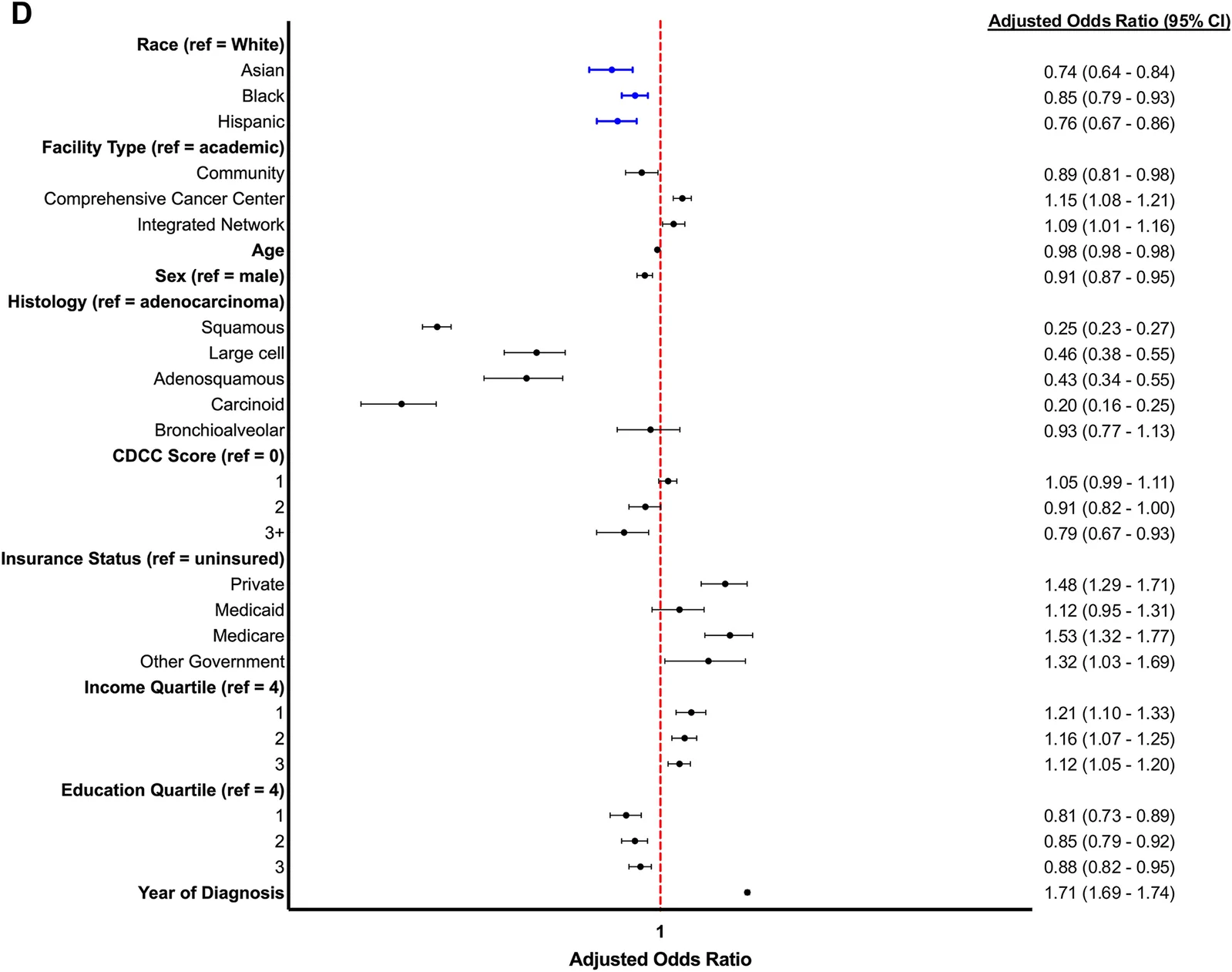
Multivariable adjusted logistic regression evaluating independent predictors of the receipt of immunotherapy for patients diagnosed with metastatic non-small cell lung cancer between 2004 and 2015 who (A) lived in high-income areas, had insurance, and were treated at academic facilities; (B) had insurance, were treated at academic facilities, and were either Black and lived in high-income areas or White and lived in low-income areas; (C) had no comorbidities, had insurance, and were treated at academic facilities; and (D) received either chemotherapy or chemoimmunotherapy. (CDCC, Charlson/Deyo comorbidity class; ref, reference.)

Disparities persisted across prespecified sensitivity analyses. Among insured patients treated at academic centers and residing in the highest income areas, Black patients remained less likely to receive immunotherapy (Figure 2A). When the cohort was restricted to insured patients treated at academic facilities, Black patients living in the highest income areas were less likely to receive immunotherapy than White patients living in the lowest-income areas (OR, 0.63; 95% CI, 0.42-0.96; Figure 2B). Similar findings were observed for patients without comorbidities (Figure 2C) and for those who received chemotherapy (Figure 2D) during the clinical trial period, indicating that disparities were present even for patients most likely to be eligible for trial participation.

**Figure 3:**
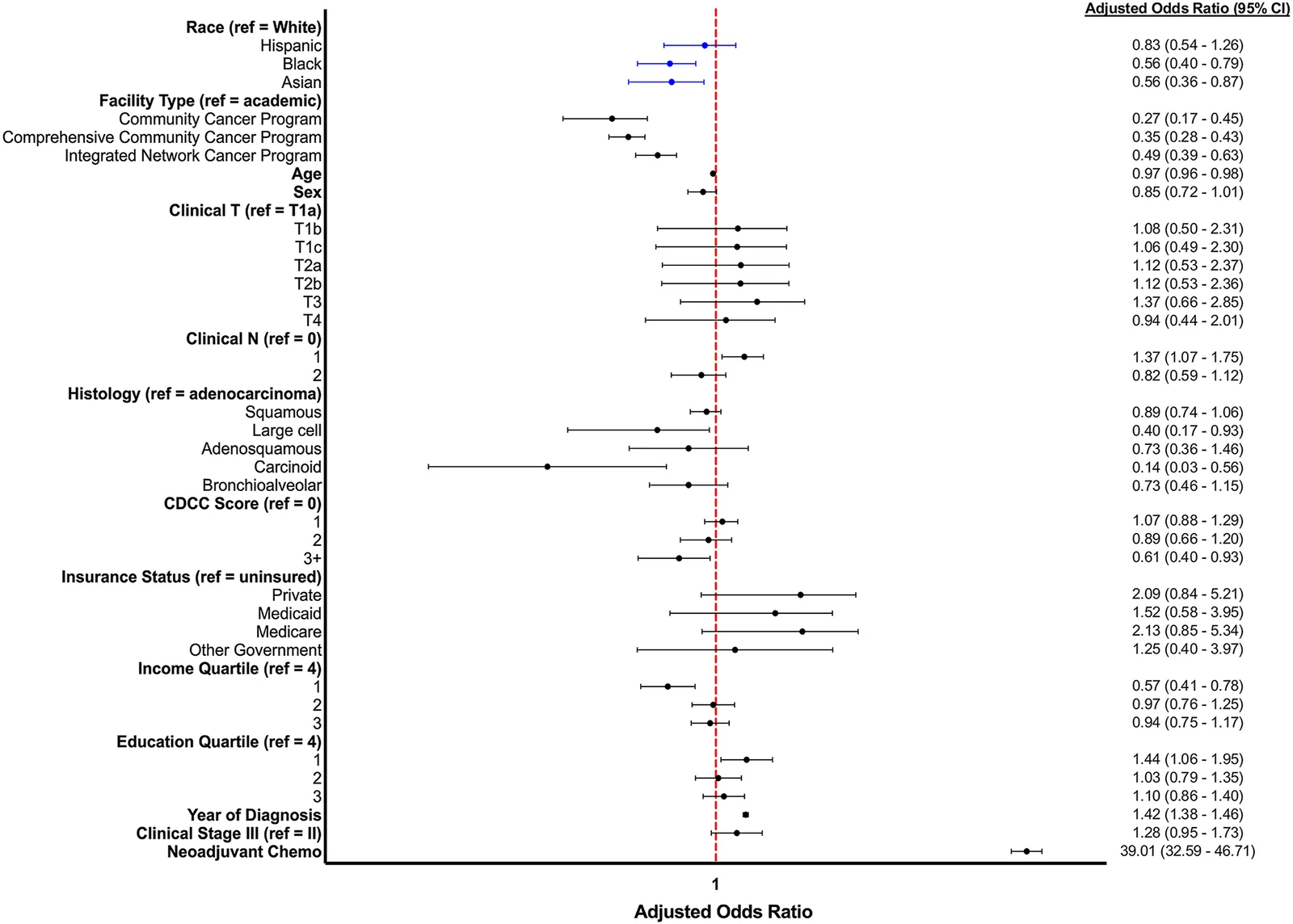
Multivariable adjusted logistic regression evaluating independent predictors of the receipt of neoadjuvant immunotherapy for patients diagnosed with resectable stage I-IIIB non-small cell lung cancer between 2004 and 2021. (CDCC, Charlson/Deyo comorbidity class; ref, reference.)

In the resectable cohort, 649 patients with stage II-IIIB NSCLC received neoadjuvant immunotherapy during the clinical trial period, of whom 86% were White, 6.2% Black, 4.0% Hispanic, and 3.7% Asian (Supplemental Table 1). In multivariable adjusted analysis, Black (OR, 0.56; 95% CI, 0.40-0.79) and Asian (OR, 0.55; 95% CI, 0.36-0.87) patients had lower odds of receiving neoadjuvant immunotherapy compared with non-Hispanic White patients (Figure 3). Lower income and treatment at nonacademic facilities were also associated with reduced likelihood of immunotherapy receipt (Figure 3).

## Comment

In this national analysis of NSCLC patients treated during periods when immunotherapy was largely accessible through clinical trials, patients from racial and ethnic minority groups were less likely than White patients to receive immunotherapy. These disparities were observed in both metastatic and resectable disease and persisted across multiple sensitivity analyses.

Importantly, disparities persisted for insured patients, those treated at academic centers, and those residing in high-income areas. Findings were also consistent for patients without comorbidities and for those who received chemotherapy. Together, these results suggest that differences in insurance status, treatment setting, or baseline medical fitness do not fully account for observed disparities in receipt of immunotherapy during trial periods.

These findings align with prior studies demonstrating underrepresentation of racial and ethnic minority patients in immunotherapy clinical trials for other cancers and extend existing literature by evaluating receipt of immunotherapy across entire clinical trial eras for both metastatic and resectable disease.^8^, ^9^, ^10^ By examining both metastatic and neoadjuvant settings, this analysis demonstrates that disparities were present during 2 separate clinical trial eras and were not limited to a single clinical context.

Several limitations warrant consideration. Receipt of immunotherapy during trial eras was used as a proxy for trial participation and may misclassify a small number of patients treated outside of formal clinical trials. In addition, the National Cancer Database does not capture programmed death ligand 1 status, which may influence eligibility for certain immunotherapy trials and limits assessment of biologic selection factors across racial and ethnic groups. The database also does not capture patients who were offered but declined trial participation and does not allow assessment of referral patterns, trial availability, or exclusion criteria by race or ethnicity.

Despite these limitations, this study demonstrates persistent disparities in access to immunotherapy during clinical trial periods for NSCLC. These findings highlight the need for concerted efforts to build trust in clinical trials and to offer novel therapies to patients from racial and ethnic minorities during early phases of clinical adoption.
