# Supplementary material for: Underrepresentation of Patients From Racial and Ethnic Minorities in Immunotherapy Clinical Trials for Non-Small Cell Lung Cancer
Source: Ann Thorac Surg Short Rep. 2026 May 20;4(3):895–902. doi: 10.1016/j.atssr.2026.03.019 (PMC13555427; doi:10.1016/j.atssr.2026.03.019)
Supplement: Supplementary file 1 [file mmc1.docx]

**Supplemental Table 1. Baseline characteristics of patients with resectable stage I-IIIB NSCLC diagnosed in the clinical trial period between 2004 and 2021**

|  | **No Neoadjuvant**  **Immunotherapy**  **(N=292,344)** | **Neoadjuvant**  **Immunotherapy**  **(N=649)** | ***P*** |
| --- | --- | --- | --- |
| Age (years), median (IQR) | 70 (63, 77) | 65 (59, 71) | <0.001 |
| Sex:  Male, n (%)  Female, n (%) | 160,425 (54.9%)  131,919 (45.1%) | 352 (54.2%)  297 (45.8%) | 0.74 |
| Race/Ethnicity, n (%) |  |  | <0.001 |
| Non-Hispanic White | 236,891 (81.0%) | 557 (85.8%) |  |
| Hispanic | 17,496 (6%) | 26 (4.0%) |  |
| Black | 31,182 (10.7%) | 40 (6.2%) |  |
| Native American | 781 (0.3%) | 2 (0.3%) |  |
| Asian | 5,994 (2.1%) | 24 (3.7%) |  |
| Charlson/Deyo comorbidity score, n (%) |  |  | <0.001 |
| 0 | 160,796 (55.0%) | 404 (62.2%) |  |
| 1 | 81,779 (28.0%) | 166 (25.6%) |  |
| 2 | 32,212 (11.0%) | 54 (8.3%) |  |
| 3+  Tumor size (mm), median (IQR)  Tumor location, n (%)  Right upper lobe  Right middle lobe  Right lower lobe    Left upper lobe  Left lower lobe  Unknown  Histology score, n (%)  Adenocarcinoma  Squamous  Large Cell  Adenosquamous  Neuroendocrine  Bronchioalveolar  Insurance status, n (%)  Not Insured  Private  Medicaid  Medicare  Other Government  Facility Type, n (%)  Community cancer program  Comprehensive community program  Academic/research program  Integrated network cancer program | 17,557 (6.0%)  46.0 (31.0,62.0)  91,006 (31.1%)  11,592 (4.0%)  49,003 (16.8%)  70,021 (24.0%)  35,935 (12.3%)  34,787 (11.9%)  123,379 (42.2%)  139,792 (47.8%)  8,230 (2.8%)  5,138 (1.8%)  6,680 (2.3%)  9,125 (3.1%)  6,591 (2.3%)  67,836 (23.2%)  17,627 (6.0%)  194,573 (66.6%)  5,717 (2.0%)  24,831 (8.5%)  125,591 (43.0%)  84,541 (28.9%)  57,381 (19.6%) | 25 (3.9%)  47.0 (29.0, 63.0)  219 (33.7%)  19 (2.9%)  120 (18.5%)  164 (25.3%)  92 (14.2%)  35 (5.4%)  361 (55.6%)  250 (38.5%)  6 (0.9%)  9 (1.4%)  2 (0.3%)  21 (3.2%)  5 (0.8%)  266 (41.0%)  39 (6.0%)  330 (50.8%)  9 (1.4%)    17 (2.6%)  123 (19.0%)  419 (64.6%)  90 (13.9%) | 0.79  0.51    <0.001  <0.001      <0.001 |
| Distance to facility (miles), median (IQR) | 9.7 (4.3, 23.4) | 17.9 (7.9, 50.3) | <0.001 |
| Zip-code income quartile, n (%) |  |  | <0.001 |
| 1  2  3  4 | 61,574 (21.1%)  74,508 (25.5%)  73,489 (25.1%)  82,773 (28.3%) | 76 (11.7%)  146 (22.5%)  164 (25.3%)  263 (40.5%) |  |
| Zip-code education quartile, n (%) |  |  | <0.001 |
| 1 | 60,174 (20.6%) | 140 (21.6%) |  |
| 2 | 87,651 (30.0%) | 173 (26.7%) |  |
| 3 | 89,343 (30.6%) | 202 (31.1%) |  |
| 4 | 55,176 (18.9%) | 134 (20.6%) |  |
| Year of diagnosis, median (IQR) | 2013 (2009, 2017) | 2019 (2018, 2020) | <0.001 |
| Received chemotherapy, n (%)  Clinical Stage  II  III  Clinical N Stage, n (%)  0  1  2  Clinical T Stage, n (%)  T1a  Tab  T1c  T2a  T2b  T3  T4 | 6,098 (2.1%)  103,498 (35.4%)  188,846 (64.6%)  121,577 (41.6%)  50,158 (17.2%)  120,609 (41.3%)  2,990 (1.0%)  15,137 (5.2%)  17,688 (6.1%)  27,605 (9.4%)  45,261 (15.5%)  81,461 (27.9%)  102,202 (35.0%) | 398 (61.3%)  223 (34.4%)  426 (65.6%)  231 (35.6%)  170 (26.2%)  248 (38.2%)  9 (1.4%)  54 (8.3%)  48 (7.4%)  75 (11.6%)  83 (12.8%)  213 (32.8%)  167 (25.7%) | <0.001  0.58  <0.001  <0.001 |

**Supplemental Table 2. Patients with resectable stage I-IIIB NSCLC diagnosed in the clinical trial period between 2004 and 2021 who were excluded from multivariable-adjusted logistic regression due to missing data**

|  | **No**  **Immunotherapy** | **Received**  **Immunotherapy** |
| --- | --- | --- |
| Age, n (%) | 0 (0.0%) | 0 (0.0%) |
| Sex, n (%) | 0 (0.0%) | 0 (0.0%) |
| Race/Ethnicity, n (%) | 12,133 (2.5%) | 220 (1.9%) |
| Histology, n (%) | 101,637 (26.0%) | 1,317 (12.5%) |
| Charlson-Deyo Comorbidity Score, n (%) | 0 (0.0%) | 0 (0.0%) |
| Insurance Status, n (%) | 9,423 (2.0%) | 207 (1.8%) |
| Year of diagnosis, n (%) | 0 (0.0%) | 0 (0.0%) |
| Facility type, n (%) | 3,700 (0.8%) | 128 (1.1%) |
| Zip-code income quartile, n (%) | 40,397 (9.0%) | 1,622 (15.9%) |
| Zip-code education quartile, n (%) | 39,902 (8.8%) | 1,602 (15.7%) |

**Supplemental Table 3. Patients with metastatic NSCLC diagnosed in the clinical trial period between 2004 and 2015 who were excluded from multivariable-adjusted logistic regression due to missing data**

|  | **No**  **Immunotherapy** | **Received**  **Immunotherapy** |
| --- | --- | --- |
| Age, n (%) | 0 (0.0%) | 0 (0.0%) |
| Sex, n (%) | 0 (0.0%) | 0 (0.0%) |
| Race/Ethnicity, n (%) | 7,849 (2.1%) | 16 (2.1%) |
| Histology, n (%) | 47,082 (13.8%) | 15 (1.9%) |
| Charlson-Deyo Comorbidity Score, n (%) | 0 (0.0%) | 0 (0.0%) |
| Insurance Status, n (%) | 5,575 (1.5%) | 0 (1.3%) |
| Year of diagnosis, n (%) | 0 (0.0%) | 0 (0.0%) |
| Facility type, n (%) | 1,788 (0.5%) | 10 (1.3%) |
| Zip-code income quartile, n (%) | 41,800 (12.0%) | 103 (14.9%) |
| Zip-code education quartile, n (%) | 39,902 (8.8%) | 101 (14.5%) |
